# Preparation and Response to the 2014 Ebola Virus Disease Epidemic in Nigeria—The Experience of a Tertiary Hospital in Nigeria

**DOI:** 10.1371/journal.pone.0165271

**Published:** 2016-10-27

**Authors:** Dimie Ogoina, Abisoye Sunday Oyeyemi, Okubusa Ayah, Austin Onabor A, Adugo Midia, Wisdom Tudou Olomo, Onyaye E. Kunle-Olowu

**Affiliations:** 1 Department of Internal Medicine, Niger Delta University/Niger Delta University Teaching Hospital, Yenagoa, Bayelsa State, Nigeria; 2 Department of Community Medicine, Niger Delta University/Niger Delta University Teaching Hospital, Yenagoa, Bayelsa State, Nigeria; 3 Department of Nursing Services, Niger Delta University Teaching Hospital, Okolobiri, Bayelsa State, Nigeria; 4 Department of Pharmacy, Niger Delta University Teaching Hospital, Okolobiri, Bayelsa State, Nigeria; 5 Department of Paediatrics, Niger Delta University/Niger Delta University Teaching Hospital, Yenagoa, Bayelsa State, Nigeria; University of Southern California, UNITED STATES

## Abstract

**Introduction:**

The 2014 Ebola Virus Disease (EVD) outbreak elicited global attention and challenged health systems around the world, Nigeria inclusive. We hereby report the preparation and response to the outbreak by a tertiary teaching hospital in Bayelsa State, Nigeria.

**Method:**

Between 4th August and 31st October 2014, we conducted a mixed cross sectional and qualitative study to ascertain the EVD-related fear, myths and misconceptions among healthcare workers (HCWs), and to evaluate the plans, activities and challenges faced by the hospital during the outbreak. Data was collected using a self-administered questionnaire as well as by documented observations during the outbreak. HCWs were asked to rate their fear of EVD from 1 (no fear) to 10 (highest fear).

**Results:**

Out of 189 respondents, majority (>75%) reported uncertainty about the myth that EVD can be prevented by drinking salt water or eating Garcinia kola, while 82% of respondents believed that EVD can be prevented by avoiding crowded places. About 40% of respondents expressed fear ratings of EVD of ≥ 7 out of 10. In response to the outbreak, the hospital established an EVD response team, organised EVD-sensitization and training programmes and commenced routine EVD surveillance activities. An EVD-isolation ward was constructed from an existing ward, a field incinerator was designed, hand sanitizers were produced locally and personal protective equipment were procured. No case of EVD was reported in the hospital, although three false alarms caused panic. Some HCWs adopted overly protective and avoidance behaviours, but these behaviours were abandoned after the outbreak was declared over.

**Conclusion:**

Our results suggest that the fear, myth and misconceptions were common among HCW during the outbreak. The EVD outbreak, however, helped to strengthen gaps in infection control and emergency preparedness in the hospital. Strategies to allay fear are required to contain future outbreaks of EVD in Nigeria hospitals.

## Introduction

The 2014 Ebola virus Disease (EVD) outbreak exposed the level of epidemic unpreparedness of countries, hospitals and healthcare workers (HCW) around the world.[1] Nigeria was declared EVD free in October 2014, after reporting 20 cases and 8 deaths from EVD.[2] The EVD cases were managed in treatment centres situated in Lagos and Port Harcourt, Nigeria.[2,3] All states in Nigeria were directed by the Federal Government to establish EVD treatment centres and to prepare for the detection, investigation and referral/management of confirmed and suspected EVD cases.[4]

The national and public health responses to the 2014 EVD outbreak in Nigeria have been described by various authors.[2,3,5] Nigeria was commended by various stakeholders for the prompt and effective response to the 2014 EVD outbreak through country-wide efforts that included strong political leadership and support, engagement of all levels of healthcare systems and international partnership.[4] However, the EVD outbreak in Nigeria was not without challenges. The behavioural and emotional responses to the 2014 EVD outbreak in Nigeria have been previously described.[6] The myth of drinking and bathing with salt water to prevent EVD was rife during outbreak in Nigeria, reportedly leading to hospitalisation of 20 persons and death of two others.[7] The media in Nigeria reported protests against proposed location of EVD treatment centres, [8] and patients being abandoned on account of fear of EVD.[9] There were misconceptions about EVD transmission leading to complete avoidance of hand shaking and crowded places by the general population.[6] Healthcare workers reportedly complained about lack of facilities for safe patient care and various studies from Nigeria during and after the outbreak have reported poor EVD-related knowledge and practice among healthcare workers.[10–12]

While many studies have described the national response to EVD in Nigeria, [2,3,5] as well as the EVD-related knowledge and attitude among healthcare workers, [10–12] to our knowledge, studies describing the preparation and responses to EVD outbreak among hospitals in Nigeria are lacking.

In this study we aim to describe the experience of a tertiary teaching hospital in preparing and responding to the 2014 EVD outbreak in Nigeria. Specifically, we report the preparations and activities undertaken as well as challenges faced by the hospital during the 2014 EVD outbreak; the opinions and behaviours of healthcare workers (HCW) during the outbreak; the EVD-related fear, myths and misconceptions among HCW; and the hospital’s experience with management of EVD false alarms.

It is hoped that the lessons learnt from this study will be useful to similar hospitals in Nigeria and other developing countries in preparing and responding to future epidemics.

## Method

### Study design

The study was undertaken using a mixed study design. First, in August 2014, about 2 weeks after the first case of EVD was reported in Lagos, Nigeria, we assessed EVD-related fear, myths and misconceptions among HCW using a cross sectional study design. Second, in October 2014, we used a qualitative study design to review the documented plans and activities of the hospital, as well as challenges faced during the outbreak and after establishment of the EVD treatment centre in the hospital.

### Setting

The study was undertaken in the Niger Delta University Teaching Hospital (NDUTH), a 200 bed hospital situated in Bayelsa state, South-South Nigeria. Bayelsa state adjoins Rivers state, which was one of the two states in Nigeria where confirmed cases of EVD were managed during the 2014 outbreak. The hospital is one of the two designated EVD-treatment centres in the state, providing specialist health services to people from at least 3 states in the south-south region of Nigeria. Infection control activities of the hospital are coordinated by the hospital’s infection prevention and control (IPAC) committee consisting of an infectious disease specialist, a public health physician, microbiologists and an infection control nurse, among others. However, the hospital’s response to the EVD outbreak was coordinated by an EVD-response team, whose membership included majority of the members of the hospital’s IPAC committee.

### Study participants and data collection

First, in August 2014, before the first hospital-organised EVD-sensitization workshop, we enrolled consecutive staff of the hospital who gave consent to be part of the study. The hospital had 500 staff including doctors (n = 98), nurses (n = 102) and other medical and non-medical health workers (n = 300). All study participants completed a self-administered structured questionnaire (S1 File) to assess their views about EVD-related myth and misconceptions and to rate their fear of EVD. Respondents were asked to answer ‘Agree’, ‘Disagree’ and ‘Uncertain’ to widely known myths (defined as something that many people believe but that does not exist or is false[13]) and misconceptions (defined as a belief or an idea that is not based on correct information, or that is not understood by people[14]) about prevention, transmission and treatment of EVD. Respondents were also asked; on a scale of 1 to 10, “how would you rate your fear of Ebola?” (Where 1 = no fear and 10 = highest fear).

Second, in October 2014, after Nigeria was declared EVD free, the IPAC committee and the hospital’s EVD response team appraised the hospital’s preparation and response to the outbreak based on documented plans, activities and challenges before, during and after the establishment of the EVD treatment centre in the hospital. We appraised the hospital’s EVD response using the WHO consolidated Ebola preparedness checklist[15] as well as the standard operating procedures on Ebola response as provided by the Federal Ministry of Health, Nigeria [16] and the WHO[17]. Specifically, we appraised coordination of the response, establishment of EVD-response committee, education and training of HCWs, provision of IPC resources and isolation precautions, as well as screening and management of suspected EVD patients.

We documented the opinions of HCW during the outbreak through focus group discussions undertaken during EVD-education and training programmes. All members of the EVD response team also documented the views and behaviour of HCW during individual discussions and during evaluation of patients referred to the EVD-response team as suspected EVD cases. All documented opinions and behaviour were reviewed and domains relating to: participation in the EVD response; screening and case management of suspected cases; use and demand for IPC resources; response to establishment of EVD-isolation ward; and post-outbreak behaviour, were identified. To report the experience in the management of EVD false alarms, we also reviewed the clinical history and management of all patients referred to the EVD-response team as suspected EVD cases.

Opinions and behaviours of HCW in the hospital were documented during hospital-based EVD sensitization programmes and during personal or group interactions with the hospital EVD’s response team. The EVD-response team also documented the experience faced with managing false alarms of EVD in the hospital.

Ethical approval for the study was obtained from the NDUTH institutional ethical review committee. Written consent was obtained from all study participants as approved by the ethical review committee.

### Statistical analysis

Study variables were summarised in frequencies and percentages. Professional differences in respondent’s opinion about EVD-related myths, misconceptions and fear were determined by chi-square and fisher’s exact test as appropriate. P<0.05 was considered statistically significant.

## Results

### Plans and activities of the hospital in response to the EVD outbreak

Two weeks after first case of EVD was reported in Lagos, the hospital’s management in conjunction with the IPAC committee immediately put together plans and activities to prevent and control EVD in the hospital. An EVD rapid response team was inaugurated consisting of some members of the IPAC committee and other volunteers from different disciplines. The EVD response team was mandated to coordinate the hospital preparations and response to the outbreak.

Hospital-wide EVD sensitization workshops were organised on various days to inform and educate staff about EVD. Thereafter, clinicians were trained on various aspects of standard precautions of infection control, healthcare waste management and clinical management of EVD, among others. There were practical demonstrations on hand hygiene and use of personal protective equipment (PPE). Infrared thermometers were procured for temperature measurements in various clinical areas. An EVD-screening and triaging checklist was developed according to national guidelines[18] and distributed to all clinical areas. All HCW were asked to be vigilant and to immediately notify hospital’s EVD response team of any suspected case.

### Creation of EVD Isolation ward

**Fig 1:**
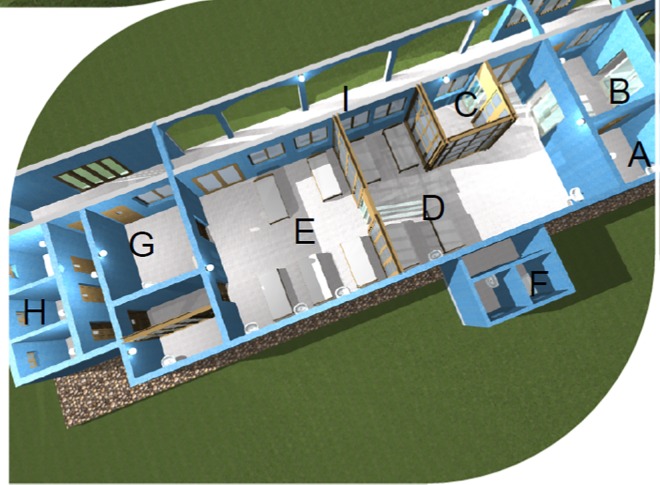
Ebola Isolation ward. The design of the isolation ward included the following- A-anteroom, B-lobby, C-triage room, D-ward for suspected cases; E-ward for confirmed cases; F- toilets/bathrooms for suspected cases; G-decontamination room; H-toilet/bathroom for confirmed cases.

Having designated the hospital as one of the state’s Ebola treatment centres, the state government provided funds for redesign of an existing ward to be used as isolation wards for admission and management of suspected and confirmed cases of EVD. The isolation wards were completed about six weeks into the outbreak and consisted of a single anteroom, a decontamination room, a separate four bed ward for suspected cases and another four bed ward for confirmed cases (Fig 1). The wards were naturally ventilated without provision for negative pressure ventilation or HEPA filters.

### Provision of infection control resources

**Fig 2:**
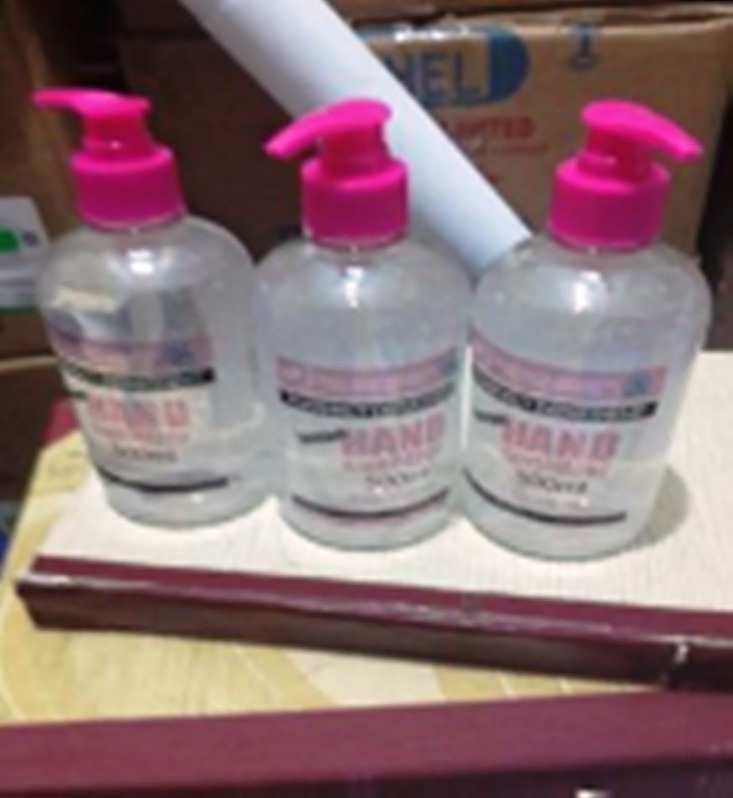
Samples of hand sanitizers produced locally by the hospital during the Ebola outbreak.

**Fig 3:**
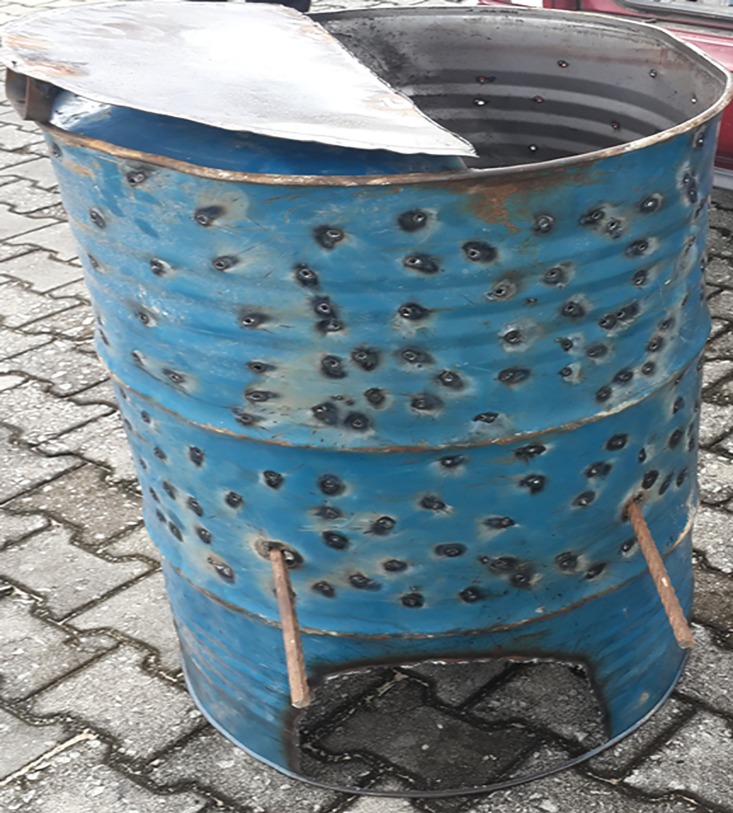
Field Incinerator. Locally made incinerator, improvised from a 220 litre drum based on WHO guidelines.

With support and collaboration of the Bayelsa State Ebola Task Force, the hospital procured full body PPEs including gowns, aprons, face shields, hand gloves, goggles, and boots. Alcohol-based hand sanitizers were initially purchased but later produced by the hospital’s pharmacy department (Fig 2) using the WHO guideline.[19] In view of absence of a hospital incinerator, the waste management team of the hospital designed a field or make-shift incinerator (Fig 3) improvised from a 220 litre drum according to WHO guidelines to be used for managing waste of suspected EVD patients.[20]

### EVD-related fear, myths and misconceptions

A total of 189 respondents, including 70 doctors, 61 nurses and 58 other medical and non-medical hospital staff, completed the questionnaire. The number (%) of healthcare workers who reported EVD-related myths and misconceptions are shown in Tables 1 and 2. About 92% of those that responded were uncertain about the role of bathing/drinking salted water in preventing EVD while 75.3% of HCW were uncertain about the preventive role of Garcinia kola (called bitter kola in Nigeria). About 82% of respondents were in agreement with the misconception that EVD can be prevented by avoiding crowded places while 59.6% of the respondents agreed with misconception that every patient with fever should be treated as a suspected case of EVD. Compared to other categories of HCW, non-medical HCW were more likely to agree that eating bitter kola is effective in preventing EVD (p = 0.014, X^2^ = 16.04, df = 6, Table 1), and also more likely to be uncertain about the role of avoiding crowded places in preventing EVD compared to other HCW (p = 0.001, X^2^ = 22,7, df = 6, Table 2). Opinions about other EVD-related myths and misconceptions were not significantly different between professional categories (p>0.05, Tables 1 and 2).

**Table 1:** Healthcare Workers’ Opinions about Common Ebola-related Prevention Myths.

Myths	Response	Doctor	Nurse	Other Health/Paramedicals	Non-Medical Health Workers	Total
**Ebola can be prevented by drinking or bathing with salt water**	Agree	0	1 (1.7%)	0	0	1 (0.5%)
	Disagree	3 (4.3%)	5 (8.3%)	2 (7.1%)	4 (15.4%)	14 (7.7%)
	Uncertain	66 (95.7%)	54 (90%)	26 (92.9%)	22 (84.6%)	168 (91.8%)
	Total	69 (100%)	60 (100%)	28 (100%)	26 (100%)	183 (100%)
**Ebola can be prevented by eating bitter kola**	Agree	1(1.4%)	5(8.3%)	1(3.6%)	3(12.0%)	10(5.5%)
	Disagree	7(10.1%)	16(26.7%)	4(14.3%)	8(32.0%)	35(19.2%)
	Uncertain	61(88.4%)	39(65.0%)	23(82.1%)	14(56.0%)	137(75.3%)
	Total	69 (100%)	60 (100%)	28 (100%)	26 (100%)	183 (100%)

**Table 2:** Healthcare Workers’ Opinions about Common Ebola-Related Prevention Misconceptions.

**Misconception**	Response	Doctor	Nurse	Other Health/Paramedicals	Non-Medical Health Workers	Total
**Every patient with fever should be treated as a suspected Ebola case**	Agree	46 (66.7%)	35 (58.3%)	15 (55.6%)	13 (48.1%)	109 (59.6%)
	Disagree	11 (15.9%)	13 (21.7%)	5 (18.5%)	4 (14.8%)	33 (18.0%)
	Uncertain	12 (17.4%)	12 (20.0%)	7 (25.9%)	10 (37.0%)	41 (22.4%)
	Total	69 (100%)	60 (100%)	27 (100%)	27 (100%)	183 (100%)
**Ebola can be prevented by avoiding crowded places**	Agree	60 (88.2%)	49 (83.1%)	21 (77.8%)	16 (61.5%)	146 (81.1%)
	Disagree	5 (7.4%)	7 (11.9%)	5 (18.5%)	2 (7.7%)	19 (10.6%)
	Uncertain	3 (4.4%)	3 (5.1%)	1 (3.7%)	8 (30.8%)	15 (8.3%)
	Total	68 (100%)	59 (100%)	27 (100%)	26 (100%)	180 (100%)
**Ebola can be prevented by avoiding shaking hands**	Agree	52 (76.5%)	43 (71.7%)	14 (51.9%)	16 (59.3%)	125 (68.7%)
	Disagree	7 (10.3%)	8 (13.3%)	9 (33.3%)	5 (18.5%)	29 (15.9%)
	Uncertain	9 (13.2%)	9 (15.0%)	4 (14.8%)	6 (22.2%)	28 (15.4%)
	Total	68 (100%)	60 (100%)	27 (100%)	27 (100%)	182 (100%)

**Fig 4:**
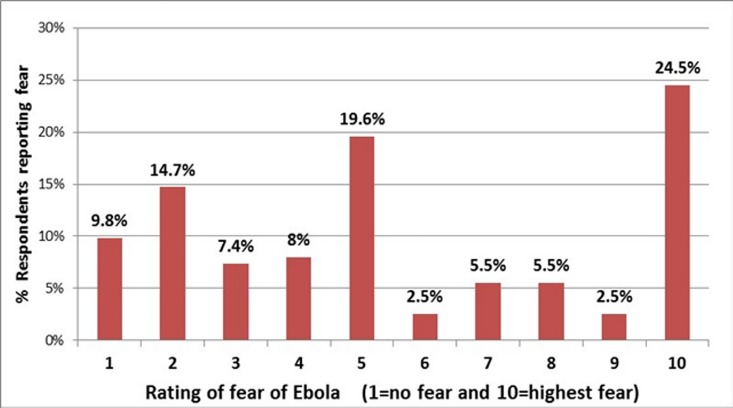
Rating of fear of Ebola by healthcare workers. More than 40% of respondents rated their fear of EVD greater or equal to 7 out of 10.

The rating of fear of EVD by respondents is shown in Fig 4. Out of 163 respondents, 40 (24.5%) rated their fear of EVD 10 out of 10 (highest level of fear) while 32 (19.6%) rated their fear 5 out of 10 (average level of fear). About 40% of respondents expressed fear ratings of EVD of greater or equal to 7 out of 10. There was no professional difference in rating of fear (p>0.05, Table 3).

**Table 3:** Professional Differences in Rating of Fear of Ebola.

Health worker category	Number of respondents	Mean fear score	Median fear score	25^th^ percentile	75^th^ percentile
**Doctor**	70	5	5	2	9
**Nurse**	61	6	5	3	9
**Other Health/Paramedicals**	29	6	5	4	8
**Non-Medical Health Workers**	29	6	5	2	10
**Total**	189	6	5	3	9

There were no significant professional differences in rating of fear (median fear scores did not differ across professional categories p>0.05, Kruskal Wallis test).

### Healthcare workers’ opinions and behaviour

Following nomination of persons to serve in the hospital’s EVD response team, it was observed that some HCW were reluctant to be part of the hospital EVD response team and some requested for life insurance and monetary stipends to participate in the EVD response. About two weeks after the first case of EVD was reported in Lagos in July 24^th^ 2014, the EVD response team received reports about HCW who avoided seeing patients with fever, who wanted to wear gloves to examine all patients irrespective of their illness and who washed their hands frequently even without patient contact. Due to excessive demand, hand gloves, sanitizers, and hand washing soaps quickly ran out of stock. When the isolation wards were being constructed, some HCW initially complained about the location of treatment centre within the hospital premises, requesting for its relocation outside the hospital. Some HCW even resolved not to visit or work in the isolation wards once completed. Many clinicians complained of lack of skills to protect themselves against contracting the infection and others were doubtful about the capacity of the hospital to adequately treat them if they were infected.

### Experience with false alarms of EVD

The hospital reported three cases of false alarms erroneously labelled as suspected cases of EVD by admitting clinicians. The first case was a 27year man who presented with fever, jaundice and yellow coloured urine, later confirmed as a case of viral hepatitis. The second was a 54year old known HIV-infected patient with cerebral toxoplasmosis who had a seizure with bleeding from the mouth due to tongue biting. The third was a 58year old man who had liver cirrhosis with haematemesis. Although none of these patients met the case definition of a suspected case of EVD, they were all stigmatized and avoided before review and exclusion of EVD by members of the EVD response team.

### Linkage with state’s EVD task force and international health organisations

The hospital had direct linkage with the state’s EVD task force as well as representatives of international organisations such as the World Health Organisation. In view of absence of an EVD diagnostic laboratory in the state, collection and transportation of laboratory specimen for any suspected case was coordinated by the state’s EVD task force in collaboration with other partners.

### Post-Outbreak Observations

By October 2014, after Nigeria was declared EVD free, the EVD response team observed that some HCW became less interested in regular hand hygiene and other IPAC activities. Demand for hand sanitizers and PPE declined dramatically. Funding for all infection control resources from the hospital, the state government and related partners also declined. Consequently, it was observed that the initial surge in IPAC activities and resources were not sustained.

## Discussion

A number of studies from around the world revealed that hospitals around the world were either unprepared or only partially prepared to respond to the 2014 EVD outbreak[21–27] One of these surveys also indicated that hospitals in African countries appeared to be less prepared than their counterparts in developed countries.[28] In agreement, we found that before the outbreak, our hospital, situated in the Niger Delta region of Nigeria, was neither adequately prepared nor equipped with necessary resources to respond to the outbreak. However, with the report of the first case of EVD in Nigeria, it is noteworthy that national and state stakeholders in Nigeria made concerted efforts to ensure that hospitals were adequately prepared. Our results also indicate that concerted efforts by the hospital, with support from other stakeholders, improved the hospital’s level of EVD’s preparedness. For instance, the hospital, for the first time in all cases, established an EVD isolation ward, produced hand sanitizers locally and provided full body PPE’s. The EVD outbreak therefore helped to strengthen the infection control activities and epidemic preparedness of the hospital.

Our results suggest that majority of HCW were uncertain about the truthfulness or otherwise of the myth that EVD can be prevented by bathing or drinking salt water and eating bitter kola. Further, majority of the HCW were in agreement with misconceptions that EVD can be prevented by avoiding hand shaking and crowded places. These myths and misconceptions might have been partly fuelled by poor knowledge and understandings of EVD, since the views of HCW were sought at the early phases of the outbreak before sensitization and training workshops. While EVD preventive information states that the disease can be prevented by avoiding physical contact with sick or suspected Ebola patients, this information has been misconceived to mean that to prevent Ebola all forms of physical contact with everybody should be avoided including hand shaking and body to body contact as may occur in crowded places. Ebola-related misconceptions were also reported in surveys conducted in other West Africa countries such as Guinea and Ghana, where respondents had varying views about aetiology of Ebola including Ebola being a spiritual condition that can be cured by praying to God, that it could be transmitted through air, mosquito bites and flies, and that Ebola epidemics was due to Western bioterrorism experiments.[29,30]

Fear might also be responsible for the emergence of myths and misconceptions about EVD.[6,31] During the 2014 EVD outbreak and other Ebola epidemics before it, fear was always reported as a common emotion among the general population and HCW within and beyond affected countries[32–35] In our study, with about 40% of respondents expressing fear ratings of EVD of greater or equal to 7 out of 10, fear possibly played a major role in the expressed negative opinions and behaviours by HCW. Fear was also partly responsible for the number of false alarms of EVD reported in the hospital. Virtually all states in Nigeria had to contend with EVD false alarms during the epidemic.[36] Although only few cases of false alarms of EVD were seen and reviewed in our hospital, our experience with these cases may suggest that during EVD outbreaks patients with jaundice or mucosal bleeding due to other causes may be falsely labelled as suspected cases of EVD.

While fear has been implicated in the emergence of negative opinions and behaviour, fear might also have a positive side. It has been suggested that fear is often a stimulus for health authorities to take action during epidemics[31] and that the fear of EVD was the stimulus that encouraged all stakeholders around the world to collaboratively fight the EVD outbreak.[33]

The fear-induced avoidance behaviour reported among HCW in our study is similar to findings reported among HCW of other hospitals around the world. In a US survey, about 26% of surveyed healthcare workers were unwilling to manage cases of EVD mainly due to fear of contracting the disease.[37] About 56% of respondents in this survey felt their hospitals were not well equipped to manage cases of EVD.[37] A US hospital also reported that some healthcare workers stayed away at home pretending to be sick for fear of contracting the disease.[38] In another survey among healthcare workers in the UK, majority did not consider or were against volunteering in the Ebola outbreak in West Arica due to fear of contracting the disease, insufficient information about the disease and partner concerns, among others.[34] Following a large outbreak of EVD in 1997 (in Kikwit DRC), a study reported that HCW avoided examining patients with fever and stayed away from hospitals and EVD-isolation facilities.[39] It is evident therefore that preparing for future outbreaks of Ebola requires strategies that will combat fear of the disease, especially among frontline HCW. Such strategies must include measures that reduce the risk of contracting of infection in healthcare settings, improve understanding of the disease, promote development of curative therapy and preventive vaccines and that ultimately reduce the perception of the disease as very severe and deadly. [6]

It is noteworthy that as Nigeria was declared EVD free, our results reveal that HCW had started abandoning protective behaviours adopted during the outbreak. Furthermore, government and other partners reduced or even stopped funding and support for infection control activities under the impression that EVD was no longer a problem. One year after Nigeria was declared Ebola-free, some stakeholders have also opined that Nigeria has failed to sustain its efforts to strengthen health systems to fight future Ebola epidemics due to the perception that Ebola is no longer a threat in Nigeria.[40] As cases of EVD continue to emerge and re-emerge in adjoining West Africa countries[41] where travel and migration to and from Nigeria for business, leisure, tourism, education and other special interests continue, Nigeria certainly remains at risk of Ebola outbreaks, especially as the health systems in Nigeria gradually forget how the country successfully halted the 2014 EVD outbreak.[40]

In this study, we used documented individual reports by members of the hospital’s EVD response as basis for defining the opinion and behaviours of HCW during the outbreak. These documented reports were not standardized and therefore prone to under-reporting due to recall bias or over-reporting due to observer bias. However, it is our opinion that the reported findings from our study are largely valid as they are similar to findings reported among HCW from other countries. While our study was restricted to a single facility in Nigeria, we believe our findings provide some useful insights on how hospitals in Nigeria prepared and responded to the 2014 EVD outbreak. However, future multi-facility and multi-regional studies are necessary to corroborate our findings.

In conclusion, we found that our hospital situated in the Niger Delta region of Nigeria was neither prepared nor equipped to respond to infectious disease outbreaks. However, the Ebola outbreak helped the hospital to strengthen gaps in infection control and epidemic preparedness. The major challenges were due to EVD-related misconceptions, myths, and fear among some hospital staff, with some attendant self-protective behaviour and excessive demand for hand sanitizers, gloves and PPEs. Strategies to allay fear, to sustain infection control supplies and to generally strengthen health systems[42] are required to contain future outbreaks of EVD in Nigeria hospitals.

## Supporting Information

S1 File Study Questionnaire. (PDF)

S2 File Study Data. (SAV)
