# Supplementary material for: Preparation and Response to the 2014 Ebola Virus Disease Epidemic in Nigeria—The Experience of a Tertiary Hospital in Nigeria
Source: PLoS One. 2016 Oct 27;11(10):e0165271. doi: 10.1371/journal.pone.0165271 (PMC5082873; doi:10.1371/journal.pone.0165271)
Supplement: S1 File — (PDF) [file pone.0165271.s001.pdf]

Study Questionnaire

Study Title **“Ebola-Related Attitude Among Health Care Workers in a Tertiary Hospital in Bayelsa State, Nigeria.”**

A. Demographic data

A1	How old were you on your last birthday?	
A2	What is your Gender?	Male [] Female [] (please tick one)
A3	What is your marital status?	Married [ ]<br>Single (never married) [ ]<br>Others…………….. …………….(specify)
A4	What is your occupation?<br>(tick as appropriate)	Doctor [ ] Nurse [ ]<br>Pharmacists [ ] Laboratory scientists [ ]<br>Others (Please specify)_______________
A5	What is your rank in your profession?	……………………………(please specify)
A6	Where do you work?	…………………………(please specify)

B. Healthcare workers’ opinions about Ebola prevention

B	Concerning prevention of Ebola, please tell us if you agree, are uncertain or disagree with the following statements (please tick as appropriate)			
B1	Every patient with fever should be treated as a suspected Ebola case	Agree [ ]	Uncertain [ ]	Disagree [ ]
B2	Ebola can be prevented by avoiding crowded places	Agree [ ]	Uncertain [ ]	Disagree [ ]
B3	Ebola can be prevented by avoiding shaking hands	Agree [ ]	Uncertain [ ]	Disagree [ ]
B4	Ebola can be prevented by drinking or bathing with salt water	Agree [ ]	Uncertain [ ]	Disagree [ ]
B5	Ebola can be prevented by eating bitter kola	Agree [ ]	Uncertain [ ]	Disagree [ ]

C. How would you rate your fear of getting Ebola on a 1 to 10 scale with 1 meaning no fear at all and 10 meaning the highest level of fear? (Please only tick as appropriate)

[1] [2] [3] [4] [5] [6] [7] [8] [9] [10]

Thank you for participating in this study
